# Supplementary material for: Partners at Care Transitions: exploring healthcare professionals’ perspectives of excellence at care transitions for older people
Source: BMJ Open. 2018 Sep 19;8(9):e022468. doi: 10.1136/bmjopen-2018-022468 (PMC6150145; doi:10.1136/bmjopen-2018-022468)
Supplement: Supplementary file 1 [file bmjopen-2018-022468supp001.pdf]

Supplementary file 1:
Questions for the Clinical Leads of the high performing specialties

Introduction to explain:

- The research programme
- The data that we have used to identify their hospital specialty
- Some of the challenges of using the data
- The aim of the telephone conversation.

Questions:

- What do you think could account for your exceptional performance?

- Broadly, how does your specialty compare to xx specialties in other trusts?

  - Processes of care e.g. specific ways in which you care for patients during discharge, care transitions, or readmissions
  - Structure and resources of your department e.g. in hospital services, community services etc.
  - Patient population e.g. case mix differences
  - Can you think of any key differences in the way that your department / hospital collects or codes their readmission data compared to others?

- Is there anything you have done as a speciality / any improvement work that you have done over the last few years that could have contributed to your readmission rate performance?

- Would you consider yourself to be at an advantage or disadvantage for discharging patients home safely and preventing readmission?

- Are your patients treated on specific wards within your trust? Our aim originally was to identify high performing wards/units. Are there any wards/units or areas within your trust that you think may perform exceptionally better than others?
