# Supplementary material for: Partners at Care Transitions: exploring healthcare professionals’ perspectives of excellence at care transitions for older people
Source: BMJ Open. 2018 Sep 19;8(9):e022468. doi: 10.1136/bmjopen-2018-022468 (PMC6150145; doi:10.1136/bmjopen-2018-022468)
Supplement: Supplementary file 2 [file bmjopen-2018-022468supp002.pdf]

Supplementary file 2:

Partners At Care Transitions (PACT): Understanding excellence from a general practice perspective

Focus group / interview framework

Things to consider:

- Transitions from hospital to home rather than care home
- Consider the full multidisciplinary team including nurses / community / external teams
- We are trying to understand not only *what* you do, but also *how* that helps your team.

Ask everyone to introduce themselves

So far I've referred to patients who move from hospital to home as going through a 'transition of care' but everyone has different perspectives and uses different terminology. **What do you consider a transition of care to be?**

- When does the process start and stop for you?
- What about from a community perspective?

Everything is a bit easier when you can tell a story! Can you think of an older person that you have cared for who has frequently been in and out of hospital? If you can think of a patient that you all know then that is great, but if not your own example is absolutely fine. Briefly describe the situation to everyone. **How was this patient supported during that time?**

- If different scenarios – does anyone have different experiences of how a similar patient has been supported?
- How / why did these actions help? (what was the function of the actions?)

Can you think of a similar patient that you have successfully and appropriately managed to keep at home? **What was different about this situation and how did that help you?**

So were now going to discuss transitions more generally. **How do you identify older people who are at risk of being admitted or readmitted to hospital?**

If a risk calculation tool is used: Is this tool always effective? How do you identify the high risk patients that aren't highlighted by the tool?

- Who is involved in this?

Once an elderly patient has been discharged from hospital, how do you monitor or keep track of their health or progress?

- Is this acceptable and/or timely?
- What about from a practice nurse, reception, or community team perspective?

In an ideal world, what sort of things would you like to know and when? How would this help?

I want you now to try and think about the system that you work in. **What makes it difficult for you to care for older patients following discharge and how do you overcome these challenges?** i.e. Everyday challenges (e.g. fulfilling follow ups, lack of info about med changes etc.)

- How does that [adaptation] help – what function does it play?
- What systems or processes have you put in place to help? e.g. who reads through discharge letters? How do you find out xxx?
- In an ideal world, what might help you overcome these challenges?

As a practice what challenges do you face and how do you overcome them? e.g. inefficient system, staff shortages, periods of excessive workload.

- How does the culture within the practice help? e.g. teamwork, communication

---

How do you find out about the good and bad care that patients receive during their transitions home?

- What have you done, either as an individual or as a team, to try and prevent this from happening again?

Older patients often don't feel very well prepared for their discharge – they don't fully understand what has happened to them, they have been deconditioned while in hospital etc. **Can you think of anything that hospital staff have done to effectively prepare older patients for discharge?** e.g. information giving, self-care, signposting, something to reduce deconditioning

- How does xxxx help the patient or improve their experience (what is the function of that).
- How does xxxx make your job a little bit easier?

OR - In an ideal (but practical) world, what could hospital staff reasonably do to better prepare older patients for discharge?

- How would this improve their safety, their experience or make your job easier?

Following a discharge home, how do you try and involve older patients and their families in their care?

- How does this make it easier for you and other healthcare professionals during a transition home?

- Is there anything else you think I need to know about how your team delivers safe care and / or involves patients during a transition from hospital to home?
- Who else should I be speaking to? (Within the team, wider teams, external healthcare teams)
- Anything I should specifically explore with secondary care?

Partners At Care Transitions (PACT): Understanding excellence from a secondary care perspective.

Focus group framework

Things to consider:

- Transitions from hospital to home rather than care home
- Perspecives of the full multidisciplinary team including AHPs, pharmacists, external teams etc.
- Trying to understand not only *what* you do, but also *how* that helps your team. We will ask 'why'!
- Do you do anything different to other similar wards?

Ask everyone to introduce themselves – explain the use of the flip chart thoughout discussion.

So far I've referred to patients who move from hospital to home as going through a 'transition of care'. **What does a transition of care mean to you?**

- When does the process start and stop?
- Who is involved?

To start off with I was wondering if you could just talk to me a little bit about hospital readmissions how they impact what you do on this ward?

- Are hospital readmissions considered to be a problem?
- Do you tend to know why hospital readmissions occur?

What do you do to try and prevent hospital readmissions? Explore using prompts on page 2

How do you identify people that are at risk of readmission or who might face problems at home?

- Who is involved in this / where does the information come from?
- How does this help?
- Is this normally effective?

Discharge and discharge planning is obviouly a really big part of the transition from hospital to home and lots of things contribute to this. We are going to take a maximum of 2 minutes now to think about all the key things that contribute to a really good discharge on your ward. We will scribble these things down and then discuss in abit more detail the things that you think your team do particularly well.

What do you think contributes to a really good discharge on your ward [for an older person]? Note down on flipchart.

Which of these things do you think your team does particularly well? Highlight on the flipchart

- Explore using prompts on page 2

PROMPTS: Take each 'thing' in turn … (Remember most teams have MDTs, safety briefings etc.)

Practicalities:

- What actually happens in xxx / what does xxx look like / how do you do xxx
- Why do you think xxx is better on your ward than on others?
- Is this any different to other wards?

Function:

- What is the purpose of xxx / What does xxx do / Why do you do it like that / How does that help you xxx?
- Sometimes easier to look at the negative – what would be the consequence of not doing xxx?

What challenges do you face / what are the challenges with xxx and how do you overcome them?

What can throw a spanner in the works and how do you cope with that? How have you / could you prevent it in the first place?

How do patients and their families contribute to a good discharge? *[Usually already discussed in questions above but incase not mentioned]*

- What does that do? Why is that important?
- What are the challenges and how do you overcome them?

How do you encourage or support patients and their families to contribute?

What about patients that don't want to be involved – the more passive patients / patients that don't have family?
